# Impact of air pollution on respiratory diseases in children with recurrent wheezing or asthma

**DOI:** 10.1186/1471-2466-14-130

**Published:** 2014-08-07

**Authors:** Susanna Esposito, Carlotta Galeone, Mara Lelii, Benedetta Longhi, Beatrice Ascolese, Laura Senatore, Elisabetta Prada, Valentina Montinaro, Stefano Malerba, Maria Francesca Patria, Nicola Principi

**Affiliations:** 1 Pediatric Highly Intensive Care Unit, Department of Pathophysiology and Transplantation, Università degli Studi di Milano, Fondazione IRCCS Ca’ Granda Ospedale Maggiore Policlinico, Via Commenda 9, 20122 Milan, Italy; 2 Department of Clinical Sciences and Community Health, Università degli Studi di Milano, Milan, Italy; 3 Icahn School of Medicine at Mount Sinai, New York, NY, USA

**Keywords:** Air pollution, Asthma, NO_2_, PM_10_, Respiratory disease, Traffic-related pollutant, Wheezing

## Abstract

**Background:**

Air pollution has many negative health effects on the general population, especially children, subjects with underlying chronic disease and the elderly. The aims of this study were to evaluate the effects of traffic-related pollution on the exacerbation of asthma and development of respiratory infections in Italian children suffering from asthma or wheezing compared with healthy subjects and to estimate the association between incremental increases in principal pollutants and the incidence of respiratory symptoms.

**Methods:**

This prospective study enrolled 777 children aged 2 to 18 years (375 with recurrent wheezing or asthma and 402 healthy subjects). Over 12 months, parents filled out a daily clinical diary to report information about respiratory symptoms, type of medication used and healthcare utilization. Clinical data were combined with the results obtained using an air pollution monitoring system of the five most common pollutants.

**Results:**

Among the 329 children with recurrent wheezing or asthma and 364 healthy subjects who completed follow-up, children with recurrent wheezing or asthma reported significantly more days of fever (p = 0.005) and cough (p < 0.001), episodes of rhinitis (p = 0.04) and tracheitis (p = 0.01), asthma attacks (p < 0.001), episodes of pneumonia (p < 0.001) and hospitalizations (p = 0.02). In the wheezing/asthma cohort, living close to the street with a high traffic density was a risk factor for asthma exacerbations (odds ratio [OR] = 1.79; 95% confidence interval [CI], 1.13-2.84), whereas living near green areas was found to be protective (OR = 0.50; 95% CI, 0.31 -0.80). An increase of 10 μg/m^3^ of particulates less than 10 microns in diameter (PM_10_) and nitrogen dioxide (NO_2_) increased the onset of pneumonia only in wheezing/asthmatic children (continuous rate ratio [RR] = 1.08, 95% CI: 1.00-1.17 for PM_10_; continuous RR = 1.08, 95% CI: 1.01-1.17 for NO_2_).

**Conclusions:**

There is a significant association between traffic-related pollution and the development of asthma exacerbations and respiratory infections in children born to atopic parents and in those suffering from recurrent wheezing or asthma. These findings suggest that environmental control may be crucial for respiratory health in children with underlying respiratory disease.

## Background

Air pollution is a major public health threat, particularly in urban areas with high traffic [1-3]. Current scientific evidence has demonstrated a potential association between urban air pollutants and adverse health effects, particularly those that affect the respiratory and cardio-vascular systems [4,5]. Children, the elderly and subjects suffering from chronic disease are particularly vulnerable groups [6-8]. However, the vulnerability of children is unique, particularly very young children. First, because the lungs of a child are still growing and early exposure to environmental pollutants can easily alter lung development and function; second, children, particularly the preschoolers, are considerably more physically active than all categories of teenagers and adults [9] with longer periods of increased breathing rate that could lead to the deposition of large amounts of environmental pollutants in the respiratory tract; third, young children are predominantly oral breathers, which means that the nasal filter is by-passed, permitting the entry of a large number and variety of pollutants into the lower airways [3].

In children, exposure to traffic-related pollutants has been linked to cough, wheezing, asthma and impaired lung function [3,10-14]; however, it remains unclear whether these effects occur in the whole pediatric population or only in children who are predisposed to or are already suffering from recurrent or chronic respiratory disease. Moreover, it is not clear whether exposure to air pollution influences the risk of more frequent and/or more severe respiratory infections in the pediatric population [15,16]. To investigate the questions, the objective of the present study was to evaluate the effect of traffic-related pollution on the development of asthma exacerbations and respiratory infections in a population of Italian children with a history of recurrent wheezing or asthma compared to healthy controls. We also estimated the association between incremental increases in principal pollutants and the incidence of respiratory symptoms over a 12-month period in both study groups.

## Methods

In this prospective study, 777 children aged 2 to 18 years from the Pediatric Clinic of the Fondazione IRCCS Ca’ Granda Ospedale Maggiore Policlinico in Milan, Italy, were enrolled in the study between November and December 2011. The study protocol was approved by the Ethics Committee of the Fondazione IRCCS Ca’ Granda Ospedale Maggiore Policlinico, and the study was conducted in accordance with the standards of Good Clinical Practice. Written informed consent was obtained from the parents or legal guardians of each enrolled child, and written assent was obtained from children older than 7 years.

The study was based on two cohorts of children. The first cohort included 375 children followed at the Respiratory Disease Section of the Pediatric Clinic and was represented by all of those with a history of recurrent wheezing or asthma for whom personal and/or parents’ assent to participate to the study was obtained. Eligibility for recruitment was based on the occurrence in the medical history of at least three pediatrician-diagnosed lower respiratory tract illnesses with wheezing in a 6-month period or pediatrician-diagnosed asthma. Asthma was defined as the presence of episodes of cough, breathlessness or dyspnea together with demonstration of airflow variability (i.e., an increase in the forced expiratory volume in one second of 12% following inhalation of 400 μg salbutamol) [17]. The second cohort comprised 402 healthy children born at term. with no history of chronic underlying disease and with no history of wheezing/asthma, negative for the presence of lower respiratory tract disease at baseline, who were randomly selected and enrolled in the study during the same period among those who attended the Outpatient Clinic for minor surgery procedures.

At the time of recruitment, parents of the children in both cohorts received information orally regarding the purposes of the project and the family commitments. After signing the informed consent form, standardized, self-administered questionnaires, completed by the parents, were used to collect information about the characteristics of the family (i.e., parental education, parental smoking, family history of atopy), clinical data regarding the child (including characteristics at birth, neonatal respiratory or cardiac diseases, duration of breastfeeding, previous hospitalizations) and the characteristics of the home environment (i.e., home type and size, location and characteristics, proximity to green areas, polluted areas or high traffic roads, exposure to passive smoke). Residence in a “green area” was defined as a home within 300 m of a park. On the contrary, home sited in polluted areas or areas with high traffic were considered those homes not sited in a residential area or far no more than 500 m from a factory, a highway or an urban expressway. The questionnaire that required 10 minutes to complete was conceived by the first author (SE) in collaboration with the co-authors (CG and MFP) and pilot tested on a sample of 20 children.

The parents completed a daily clinical diary over a period of 12 months beginning in January 2012, reporting information about the type and frequency of respiratory diseases, types of medications used and healthcare utilization. Diagnosis of asthma attack was made when, in a subjects without respiratory symptoms, cough, breathlessness or dyspnea together with pediatrician-documented airflow variability suddenly occurred. To avoid recall bias, the compliance of each family was checked weekly via a telephone call. A clinical follow-up was performed approximately every two months for the whole study population.

Clinical data were combined with information collected from the air pollution monitoring system. Daily information on the concentrations of the two most common pollutants (nitrogen dioxide [NO_2_] and particulates less than 10 microns in diameter [PM_10_]) in the municipalities of Lombardy were obtained from the air quality monitoring stations managed by the Regional Environmental Protection Agency (REPA) of Lombardia (located in different points of the city of Milan and its hinterland). The number of stations for monitoring air pollutants were eight for Milan and seven for the hinterland. The methods and technologies used to measure air concentrations were those designated at national and international level. In particular, for particulate matter, PM10 were currently obtained using beta-ray attenuation monitors and corrected TEOM (Tapered Element Oscillating Microbalance). The REPA determined correction factors for particulate matter levels based on gravimetric and TEOM measure comparison. The parallel geometric instruments were placed in different sites and for different period during the year; the correction factors have been verified retrospectively and subsequently applied [18]. A modeling system was applied by REPA to compute the spatial distribution of air pollutants [19], and in our analyses we used data on air pollutants at a municipality–level. A selection of meteorological parameters, including temperature (daily average, minimum and maximum), relative humidity, precipitation and wind speed were also collected for adjustment. There were no day missing from the air pollution data during the study.

Comparisons between children with wheezing or asthma and healthy children were performed by contingency table analysis with the *χ*^2^ test for categorical variables or Wilcoxon’s rank-sum test (for data that were not normally distributed based on the Shapiro-Wilk test) for continuous variables. Odds ratios (OR) and 95% confidence intervals (CI) were calculated to measure the association between various indices of air pollution exposure and the occurrence of asthma in the group of children with wheezing/asthma. The ORs were computed using unconditional multiple logistic regression models that included terms for age, sex, number of siblings, parental education and presence of smokers at home. The association between air pollutants and episodes of lower respiratory tract infections was quantified using multivariate regression models based on the generalized estimating equations [19] to adjust for within-subject correlations due to repeated measurements in the responses. We fitted the generalized longitudinal models using the logit link and binomial errors with the GENMOD procedure in SAS.

The levels of air pollutant exposure were calculated as 3-day means (lag 0–2) of the daily weighted average PM_10_ and the maximum daily NO_2_ level in each subject’s municipality of residence. These levels were introduced in the models either as categorical variables (i.e., categories were defined a priori*,* for PM_10_ on the basis of air quality standards defined by the European Commission, that established a 24-hour average health based maximum level of 50 μg/m^3^; and for NO_2_ on the basis of tertiles of exposure) or continuous variables (i.e., results represent the increase or decrease in risk corresponding to an increment of 10 μg/m^3^ in the level of the pollutant considered). Other time-dependent covariates that were included in the multivariate analyses were temperature (3-day means), relative humidity (3-day means), day of the week, and season. Invariant covariates were sex, age at enrolment, parental education, number of siblings at enrollment and presence of smokers at home.

We computed the ORs and corresponding 95% CI for the recurrent wheezing/asthma and healthy subjects adjusting for the above variables. Because the probability of a lower respiratory tract infection was low for a given child on a given day of the study period, the OR represents a satisfactory approximation of the rate ratio (RR).

All the analyses were two-tailed, and p ≤ 0.05 was considered statistically significant. All of the analyses were conducted using SAS version 9.2 (Cary, NC, USA).

## Results

Of the 777 children who were initially recruited for the study, 84 (10.8%) were excluded from the statistical analysis due to incomplete or unavailable respiratory outcome information during the follow-up period. Consequently, 329 children with recurrent wheezing or asthma and 364 healthy subjects (with information available for at least 9 months of follow-up) were included in the study. The participants of each group excluded from the study did not differ from the included children of the same group with respect to baseline characteristics.

The sociodemographic and clinical characteristics of the participants are shown in Table 1. At baseline, the two cohorts were comparable regarding age distribution, maternal smoking during pregnancy, birth weight, duration of breastfeeding, living in proximity to a street with heavy traffic, presence of a stove/fireplace at home, and presence of pets at home. Male sex (p < 0.001), lower parental education (p < 0.001), paternal smoking during pregnancy (p < 0.001), parental history of atopy (p < 0.001), prematurity (p < 0.001), neonatal respiratory disease (p < 0.001), neonatal cardiac disease (p = 0.01), previous hospitalization for respiratory disease (p < 0.001), living in a house or villa (p = 0.004), small home size (p = 0.008), proximity to a factory (p < 0.001), residence in green areas (p = 0.046), smokers at home (p = 0.004) and absence of an air conditioning system at home (p = 0.007), were significantly more frequent in the recurrent wheezing/asthma group than in healthy children.

**Table 1:** Sociodemographic and clinical characteristics of children with recurrent wheezing or asthma and healthy children

	**Wheezing/asthma (n = 364)**		**Healthy children (n = 389)**		**p-value**
	**N**	**%**	**N**	**%**	
*Age distribution*					
2-5 yrs	155	42.6	152	39.1	
5-18 yrs	209	57.4	237	60.9	0.365
*Sex*					
Male	237	65.1	196	50.4	
Female	127	34.9	193	49.6	<0.001
*Parental education*^*a*^					
Primary/secondary for both parents	36	9.9	17	4.4	
High school for at least one parent	161	44.5	129	33.2	
University degree for at least one parent	165	45.6	243	62.5	<0.001
*Paternal smoking during pregnancy*^*a*^					
No	255	71.2	312	81.0	
Yes	103	28.8	73	19.0	0.002
*Maternal smoking during pregnancy*^*a*^					
No	329	91.6	353	91.5	
Yes	30	8.4	33	8.5	0.92
*Parental history of atopy*^*a*^					
No	269	74.9	343	90.5	
Yes	90	25.1	36	9.5	<0.001
*Gestational age (weeks)*^*a*^					
<37	56	15.6	26	6.8	
≥37	304	84.4	357	93.2	<0.001
*Birth weight (grams)*^*a*^					
≤2500	44	12.2	31	8.0	
>2500-3500	227	62.9	245	63.1	
>3500	90	24.9	112	28.9	0.11
*Neonatal respiratory disease*^*a*^					
No	317	88.1	372	95.9	
Yes	43	11.9	16	4.1	<0.001
*Neonatal cardiac disease*^*a*^					
No	335	94.4	380	97.9	
Yes	20	5.6	8	2.1	0.01
*Breastfeeding (months)*					
No breastfeeding	70	19.2	56	14.4	
≤6	152	41.8	152	39.1	
>6	142	39.0	181	46.5	0.07
*Previous hospitalization for respiratory disease*^*a*^					
No	187	52.2	339	87.4	
Yes	171	47.8	49	12.6	<0.001
*Home type*^*a*^					
Flat	318	87.8	364	93.8	
House/villa	44	12.2	24	6.2	0.004
*Home size (m*^*2*^*)*^*a*^					
≤90	155	43.9	123	32.8	
>90-110	103	29.2	127	33.9	
>110	95	26.9	125	33.3	0.008
*Proximity to car-traffic*					
No	168	46.2	169	43.4	
Yes	196	53.8	220	56.6	0.46
*Proximity to truck-traffic*					
No	304	83.5	342	87.9	
Yes	60	16.5	47	12.1	0.08
*Proximity to factories*					
No	327	89.8	375	96.4	
Yes	37	10.2	14	3.6	<0.001
*Residence in a green area*					
No	145	39.8	183	47.0	
Yes	219	60.2	206	53.0	0.046
*Smokers at home*^*a*^					
No	241	67.7	297	77.1	
Yes	115	32.3	88	22.9	0.004
*Presence of a stove/fireplace at home*^*a*^					
No	327	90.6	358	92.3	
Yes	34	9.4	30	7.7	0.41
*Presence of an air conditioning system at home*^*a*^					
No	170	47.1	145	37.4	
Yes	191	52.9	243	62.6	0.007
*Presence of dogs at home*^*a*^					
No	319	88.4	351	90.7	
Yes	42	11.6	36	9.3	0.30
*Presence of cats at home*^*a*^					
No	338	93.6	351	90.7	
Yes	23	6.4	36	9.3	0.14

^a^The sums do not add up to the total because of some missing values. p-values were calculated using the *χ*^2^ test.

The occurrence of respiratory symptoms in both cohorts during the follow-up period is shown in Table 2. In comparison with healthy children, children with recurrent wheezing or asthma reported significantly more days of fever (p = 0.005) and cough (p < 0.001), episodes of rhinitis (p = 0.04) and tracheitis (p = 0.01), asthma attacks (p < 0.001), episodes of pneumonia (p < 0.001) and hospitalizations (p = 0.02). Children with recurrent wheezing/asthma and healthy children reported a similar number of episodes of rhinosinusitis, pharyngitis, acute otitis media and laryngitis.

**Table 2:** **Respiratory symptoms during follow-up in children with recurrent wheezing or asthma and healthy children**^**a**^

**Clinical characteristics**	**Wheezing/asthma (n = 329)**		**Healthy children (n = 364)**		**p-value**
	**N**	**%**	**N**	**%**	
*Days with fever (≥37°), mean ± SD*	7.3 ± 7.3		5.9 ± 6.4		0.009
0	66	20.1	92	25.3	
1-4	87	26.4	99	27.2	
5-9	74	22.5	101	27.7	
≥10	102	31.0	72	19.8	0.005
*Days with cough, mean ± SD*	31.7 ± 29.9		17.9 ± 23.7		<0.001
0	20	6.1	81	22.2	
1-6	33	10.0	68	18.7	
7-20	107	32.5	108	29.7	
≥21	169	51.4	107	29.4	<0.001
*Rhinitis*					
0	270	82.1	321	88.2	
1	38	11.5	23	6.3	
≥2	21	6.4	20	5.5	0.04
*Rhinosinusitis*					
0	315	95.7	351	96.4	
≥1	14	4.3	13	3.6	0.64
*Pharyngitis*					
0	239	72.6	274	75.3	
1	52	15.8	56	15.4	
≥2	38	11.6	34	9.3	0.61
*Acute otitis media*					
0	263	79.9	313	86.0	
1	40	12.2	28	7.7	
≥2	26	7.9	23	6.3	0.09
*Laryngitis*					
0	304	92.4	338	92.9	
≥1	25	7.6	26	7.1	0.82
*Tracheitis*					
0	294	89.4	344	94.5	
≥1	35	10.6	20	5.5	0.01
*Asthmatic attacks*					
0	170	51.7	335	92.0	
1	63	19.1	19	5.2	
2	46	14.0	4	1.1	
≥3	50	15.2	6	1.7	<0.001
*Pneumonia*					
0	221	67.2	309	84.9	
1	61	18.5	31	8.5	
≥2	47	14.3	24	6.6	<0.001
*Hospitalizations*					
No	306	93.0	353	97.0	
Yes	23	7.0	11	3.0	0.02
*Subjects with a complete follow-up*^*a*^	306	93.0	346	95.0	0.25
*Months of follow-up, mean ± SD*^*a*^	11.9 ± 0.6		11.9 ± 0.5		0.26

^a^Analyses were based on subjects with available information for at least 9 months of follow-up. SD: standard deviation; p-values were calculated using the *χ*^2^ test or Wilcoxon’s rank-sum test, as appropriate. Parental-eport of physician-diagnosed episodes.

Table 3 summarizes the indices of exposure to air pollutants and the incidence of asthmatic episodes during follow-up in the recurrent wheezing/asthma cohort. Living close to a street with a high density of traffic was a risk factor for asthma exacerbations (OR = 1.79; 95% CI, 1.13-2.84), whereas living near green areas was protective (OR = 0.50; 95% CI, 0.31 -0.80).

**Table 3:** **Indices of exposure to air pollution and incidence of asthmatic episodes**^**a **^**during follow-up in 329 children with recurrent wheezing or asthma**^**b**^

	**Asthma episode(s) (n = 159)**		**No asthma episodes (n = 170)**		**OR (95% CI)**^**c**^
	**N**	**%**	**N**	**%**	
*Proximity to car-traffic*					
No	66	41.5	93	54.7	1 (reference)
Yes	93	58.5	77	45.3	1.79 (1.13-2.84)
*Proximity to factories*					
No	144	90.6	150	88.2	1 (reference)
Yes	15	9.4	20	11.8	0.83 (0.40-1.74)
*Residence in a green area*					
No	73	45.9	53	31.2	1 (reference)
Yes	86	54.1	117	68.8	0.50 (0.31-0.80)

^a^At least one episode during follow-up; ^b^analyses were based on subjects with available information for at least 9 months of follow-up; ^c^odds ratios (OR) adjusted for age, sex, number of siblings, parental education and presence of smokers at home.

Table 4 summarizes the multivariate RRs and 95% CIs for pneumonia events in the study population according to the levels of air pollutants. In the recurrent wheezing/asthmatic children, an increase of 10 μg/m^3^ of PM_10_ and NO_2_ increased the incidence of pneumonia (continuous RR = 1.08, 95% CI: 1.00-1.17 for PM_10_; continuous RR = 1.08, 95% CI: 1.01-1.17 for NO_2_).

**Table 4:** Multivariate rate ratios (RR) and 95% confidence intervals (CI) of pneumonia events in children with recurrent wheezing or asthma and healthy children according to air pollutant levels

	**Asthmatic subjects (329 subjects; =143 events)**	**Healthy subjects (364 subjects; n = 80 events)**
	**RR (95% CI)**^**a**^	**RR (95% CI)**^**a**^
*PM*_*10*_*(lag 0–2 days)*^*b*^		
Low (<30 μg/m^3^)	1 (reference)	1 (reference)
Intermediate (30- < 50 μg/m^3^)	1.23 (0.73-2.05)	1.47 (0.76-2.84)
High (≥50 μg/m^3^)	1.62 (0.92-2.87)	1.04 (0.46-2.33)
Continuous RR ^d^	1.08 (1.00-1.17)	0.99 (0.88-1.11)
*NO*_*2*_*(lag 0–2 days)*^*c*^		
Lowest tertile (<89 μg/m^3^)	1 (reference)	1 (reference)
Intermediate tertile (89- < 113 μg/m^3^)	1.20 (0.75-1.90)	1.45 (0.80-0.63)
Highest tertile (≥113 μg/m^3^)	1.56 (1.01-2.42)	1.24 (0.67-2.31)
Continuous RR ^d^	1.08 (1.01-1.17)	1.02 (0.93-1.12)

^a^RRs and 95% CIs were obtained from multivariate repeated-measures analyses adjusted for age, sex, education of parents, presence of smokers at home, season, day of week, temperature and relative humidity; ^b^the mean of three days (day -2 to day 0) for the daily weighted average PM_10_ in the municipality of residence of each subject; ^c^the mean of three days (day -2 to day 0) of the maximum daily level of NO_2_ in the municipality of residence of each subject. ^d^the results represent the increase (or decrease) in risk corresponding to an increment of 10 μg/m^3^ in the level of the pollutant examined.

## Discussion

The results of the present study demonstrate that exposure to traffic-related air pollution is associated with an increased risk of respiratory morbidity in children with pre-exisiting asthma or lower respiratory tract disease with wheezing. Our findings are consistent with the results of a recent prospective study in Sweden in which 4,089 children followed from birth to the age of 12 years showed a positive association between exposure to air pollution (PM_10_ and NO_2_) during early years of life and asthma exacerbations [20]. Additionally, the association between exposure to particulates and persistent wheezing during infancy was confirmed in the Cincinnati birth cohort study of more than 700 infants born to atopic parents [21]. Furthermore, a cross-sectional study conducted in China of more than 3,000 schoolchildren documented that long-term exposure to high air pollution concentrations was associated with reduction in lung function and that asthmatic children were more susceptible to this effect [22].

The finding that episodes of bronchial obstruction related to air pollution due to traffic occurs almost exclusively in children born to atopic parents or in children who have been previously diagnosed with wheezing or asthma could be clinically relevant because it could permit the early identification of subjects living in areas with traffic-related air pollution who have an increased risk for the development of respiratory problems later in life. However, this conclusion should be considered with caution for several reasons. Most of the data that lead to this conclusion, such as those regarding home environment and the risk of pollution exposure, were reported by the parents and, consequently, had debatable reliability. Moreover, to be born to atopic parents does not mean that a child is himself atopic and that has an increased risk of development of bronchial hyperresponsiveness. The analysis of the characteristics of the enrolled children revealed that the two groups of subjects were substantially different from a socio-economic point of view. The asthma cohort had parents with lower level of education and lived in less elaborate homes because smaller and more frequently not air conditioned. A number of studies has reported that socio-economic status could condition response to air pollution with the adverse effects greater in individuals in lower social classes [23-25]. Consequently, it is possible that, at least in part, the results of this study could be due to the difference in socio-economic status of patients in comparison to healthy controls.

Moreover, the period of study was relatively short and it cannot be excluded that a longer study duration could have led to different results.. It has been reported that children with long-term exposure to traffic-related air pollution can develop respiratory disease after several years [10-14]. Consequently, some of the healthy children included in the present study, particularly the youngest, could have developed bronchial obstruction after the study was concluded. Moreover, the genetic characteristics of the enrolled children, which differ from those that lead to atopy and were not considered in this study, could have reduced or increased susceptibility of the children to wheezing or asthma independently of traffic-related pollutants. The close relationship between genetic variants and increased susceptibility to bronchial obstruction has been repeatedly reported [26,27].

In the present study, the risk of developing wheezing or asthma was strictly related to the degree of exposure to traffic-related air pollutants, with fewer incidences occurring in children living in a green area compared with those living in areas with the highest degree of air pollution. This finding is consistent with the results of several studies showing greater evidence of an association between traffic-related air pollutants and respiratory problems in children living near roads with heavy traffic with a higher incidence of exacerbations with increasing concentrations of pollutants [28,29].

In this study a close association between paternal smoking during pregnancy, prematurity, respiratory and cardiac diseases at birth with recurrent wheezing or asthma was found, in agreement with previous reports [30,31]. We did not find any significant differences between the two groups with respect to the duration of breastfeeding, in contrast to the results of some studies showing a protective role of human milk in the development of asthma [32,33] and consistent with research that failed to demonstrate any long-term protection [34,35]. In our study, children with recurrent wheezing or asthma had greater exposure than healthy controls to passive smoke in their homes. It is surprising and unfortunate that despite the well-known negative effects of passive smoke on recurrent or chronic respiratory disease [3], children with recurrent wheezing or asthma continued to be exposed to this risk factor. We did not detect any differences between children with recurrent wheezing or asthma with respect to the presence of domestic pets, particularly cats and dogs. This result appeared to exclude an effect of pet ownership on respiratory symptoms and was consistent with some previous findings [36]. However, it has been reported that exposure to cats during the first year of life is an independent risk factor for wheezing and asthma at the age of 7 years [37].

In the present study, traffic-related pollutants appeared to play a relevant role in conditioning the incidence of respiratory infections. Children with atopy or recurrent wheezing or asthma presented more respiratory tract infections including pneumonia compared with control subjects. These findings are not surprising as patients with asthma are more susceptible to recurrent viral respiratory infections [38]. Allergic inflammation in the airways leads to an impaired barrier function that facilitates the penetration of infectious particulate allergens, favoring the development of inflammatory responses and impaired interferon responses to viral infection in asthmatic children irrespective of their atopic status [39,40]. However, in the current study, in children with recurrent wheezing or asthma but not in healthy controls, pneumonia events appeared to be related to the concentrations of PM_10_ and NO_2,_ clearly supporting the hypothesis that traffic-related air pollution increases the risk of respiratory infections. However, other studies have demonstrated an association between exposure to common air pollutants and susceptibility as well as severity of respiratory infections. In a recent meta-analysis, Dherani et al. documented that the risk of pneumonia nearly doubled in young children exposed to unprocessed solid fuels [41]. Gurley et al. showed that each hour of exposure to PM_2.5_ concentrations exceeding 100 μg/m^3^ was associated with a 7% increase in the incidence of acute lower respiratory infections among children aged 0–11 months [42]. Finally, a study conducted in 33,632 Italian children (aged 6–7 years) and adolescents showed that children living in polluted areas had a great risk of cough or phlegm, whereas traffic pollution was weakly associated with asthmatic symptoms [43]. To the best of our knowledge, the present study is the first to demonstrate that the effects of air pollution on the occurrence of respiratory tract infections are observed only in children with recurrent wheezing or asthma. This finding might be explained by an increase in pollution-induced airway inflammation, genetic susceptibility to oxidative stress and the role of epigenetics in lung damage induced by air pollutants [3].

A further limitation of the present study is that children suffering from recurrent wheezing and asthma were considered in the same group. The former is not a homogeneous disease, and only a few phenotypes of wheezing can be considered as asthma before the school age [44]. Limiting the analysis to older children would permit a more reliable diagnosis of asthma but would exclude the younger age group in which exposure to air pollution is known to be associated with an increased risk of subsequent asthma onset. Moreover, respiratory diseases were assessed based on a parental questionnaire, which could have been influenced by a number of subjective issues. We tried to limit the bias in responses with weekly telephone calls by medical practitioners. A final limitation is that we focused on traffic pollution in the zone of residence but did not collect information about other outdoor exposures such as those near schools.

## Conclusions

Our findings provide evidence that there is a significant association between air pollution associated with traffic and asthma exacerbations and respiratory infections in children born to atopic parents and in children suffering from recurrent wheezing or asthma. Although this association may also occur in healthy children, correlating with the duration and intensity of exposure, children with a pre-existing respiratory susceptibility seem to be more vulnerable to the adverse effects of air pollutants. Further studies are needed to confirm this finding and to evaluate whether other factors can contribute to this association.

## Abbreviations

95% CI: 95% confidence intervals; NO_2_: Nitrogen dioxide; ORs: Odds ratios; PM_10_: Particulates less than 10 microns in diameter; RR: Rate ratio.

## Competing interests

The authors declare that they have no competing interests.

## Authors’ contributions

SE designed the study and drafted the manuscript; CG and SM performed the statistical analysis; ML participated in the enrollment and follow-up of the children and in drafting the manuscript; BL, BA, LS and EP participated in the enrollment and follow-up of the children; VM supervised the data entry and management; MFP assisted with data interpretation and manuscript development; NP critically revised the study design and manuscript. All of the authors read and approved the final version of the manuscript.

## Pre-publication history

The pre-publication history for this paper can be accessed here:

http://www.biomedcentral.com/1471-2466/14/130/prepub
